# ASO Author Reflections: Cytoreductive Surgery with Hyperthermic Intraperitoneal Chemotherapy (CRS-HIPEC) of Extraperitoneal Abdominal Disease, a Likely Common Practice but Infrequently Reported

**DOI:** 10.1245/s10434-025-16968-9

**Published:** 2025-02-03

**Authors:** Christopher W. Mangieri, Edward A. Levine

**Affiliations:** Division of Surgical Oncology, Atrium Wake Forest Baptist Medical Center, Winston-Salem, USA

## Past

The genesis of peritoneal surface malignancy (PSM) surgery dates back to the late 1920s with Dr. Meig’s pioneering work evaluating cytoreductive surgery (CRS) with ovarian cancer. Advances in CRS were achieved by surgeons such as Dr. Griffith at the National Cancer Institute and Dr. Long at the University of Alabama expanding the scope of CRS. Dr. Sugarbaker is primarily accredited for combining hyperthermic intraperitoneal chemotherapy (HIPEC) with CRS into the modern practice of PSM surgery. Randomized control trials led by Dr. Verwaal and Dr. Cashin as well as the most recent PRODIGE7 trial have validated CRS-HIPEC as standard-of-care therapy for PSM. This foundational research and these landmarks trials are rooted in the premise that CRS-HIPEC is effective only when disease is limited to the peritoneal cavity.

## Present

As the practice of PSM surgery continues to evolve since the inception of CRS approximately a century ago, the principles of CRS-HIPEC have similarly progressed. One such expansion of PSM surgical research has been evaluating the utility of extraperitoneal cytoreductions with HIPEC. While thoracic CRS-HIPEC has demonstrated effectiveness for pleural mesothelioma, abdominal extraperitoneal cytoreduction (AEC) with HIPEC for the more common etiologies of carcinomatosis, such as appendiceal and colorectal cancer, has been inconclusive and rarely specifically investigated. With limited objective data to support AEC current expert PSM guidelines, such as those provided by the Chicago Consensus Working Group and the Canadian HIPEC Collaborative Group, the presence of extraperitoneal disease is proposed as a contraindication to proceeding with CRS-HIPEC.^1–3^ However, those collectives do not specifically address AEC. It is likely that AEC is routinely performed at many high-volume HIPEC centers but is underreported in the literature.

The objective of our recent study was to evaluate the long-term oncologic outcomes of an AEC performed for appendiceal and colorectal neoplasms undergoing CRS-HIPEC.^4^ We performed a retrospective review of our institutional CRS-HIPEC registry, evaluating over 860 cases, for which 101 cases underwent an AEC. For the primary study objective of long-term survival, there was no difference in 5-year survival rates for overall survival (OS) [odds ratio (OR) 1.036, 95% confidence interval (CI) 0.671–1.597, *P* = 0.874] or disease-free survival (DFS) (OR 0.894, 95% CI 0.476–1.681,* P* = 0.728) nor was there any difference in survival times for OS (*P* = 0.955) and DFS (*P* = 0.210). Multivariate Cox regression also revealed that AEC was not an independent predictor of survival for OS [hazard ratio (HR) 1.281, 95% CI 0.885–1.854, *P* = 0.190], DFS (HR 1.087, 95% CI 0.694–1.701, *P* = 0.716], or PFS (HR 0.650, 95% CI 0.243–1.738). Furthermore, performing an AEC was not significant with regard to achieving a complete cytoreduction (HR 0.838, 95% CI 0.384–1.828, *P* = 0.657). Subgroup analyses with regard to primary tumor, to include LAMN versus appendiceal adenocarcinoma, likewise revealed no significant adverse survival with performance of an AEC. Our recently published study is the largest analysis evaluating oncologic outcomes and long-term survival for CRS-HIPEC involving AEC. The only other large-volume study evaluating AEC was performed by the US HIPEC Collaborative, which retrospectively compared approximately 40 cases of AEC with over 1000 standard CRS-HIPECs published in 2022.^5^ In that study, AEC was associated with significantly inferior OS and DFS, but on propensity-score matching there was no significant difference in survival.

## Future

While the overall amount of scientific research with AEC remains sparse, our recent study combined with other contemporary publications suggests that there is no inferior survival when an AEC is performed. Moreover, it is our anecdotal experience that AEC is commonly performed and the majority of PSM surgeons would not consider the presence of extraperitoneal abdominal disease as a barrier to proceeding with CRS-HIPEC. We encourage additional reporting of AEC experience by high-volume PSM centers and HIPEC collectives. Pending additional academic validation, we propose that PSM expert guidelines support the performance of an AEC, particularly if a complete cytoreduction can be accomplished, and modify guidance regarding extraperitoneal abdominal disease as not a contraindication to CRS-HIPEC.
